# Individual differences in the experience of meta-mood and internalizing psychopathology

**DOI:** 10.1192/j.eurpsy.2022.1815

**Published:** 2022-09-01

**Authors:** D. Yildirim, J. Vives, S. Ballespí

**Affiliations:** 1 Universitat Autonoma de Barcelona, Clinical And Health Psychology, Barcelona, Spain; 2 Universitat Autonoma de Barcelona, Department Of Psychobiology And Methodology Of Health Sciences, Barcelona, Spain

**Keywords:** Anxiety, comorbidity, emotional self-awareness, Depression

## Abstract

**Introduction:**

Emotional competencies such as attention to emotion and emotional clarity have been extensively studied in the literature. Depending on the context, their role shows different patterns of association with emotion regulation and psychopathological states.

**Objectives:**

In the current study, we aim to understand when and how attention to emotion and emotional clarity are related to the co-occurrence of anxiety and depression.

**Methods:**

Data were collected on attention to emotion, emotional clarity, anxiety, and depression. A sample of 258 adolescents aged 12 to 18 years (*M* = 14.6, *SD* = 1.7, 54.5% girls) was examined to investigate the moderating role of attention to emotion and emotional clarity on the relationship between anxiety and depression after controlling for age, gender, and socioeconomic status.

**Results:**

showed that high levels of attention to emotion and low levels of emotional clarity were associated with increased risk for anxiety and depression. Balanced levels of attention to emotion and emotional clarity were also associated with increased risk for anxiety and depression. However, low levels of attention to emotion and high levels of emotional clarity showed no statistically significant association with the occurrence of anxiety and depression.

**Conclusions:**

Overall, this positive imbalance of low attention to emotion and high emotional clarity appears to be the most favorable emotional states for coping with internalizing problems, suggesting less harmful effects of attention to emotion.

**Disclosure:**

No significant relationships.

